# Early quality-of-life and psychological predictors of disease-free time and survival in localized prostate cancer

**DOI:** 10.1007/s11136-018-2069-z

**Published:** 2018-12-03

**Authors:** Ulla-Sisko Lehto, Markku Ojanen, Anna Väkevä, Tadeusz Dyba, Arpo Aromaa, Pirkko Kellokumpu-Lehtinen

**Affiliations:** 1 Medical School, Oncology, University of Tampere, 33014 Tampere, Finland; 2 Department of Psychology, University of Tampere, 33014 Tampere, Finland; 3 Finnish Cancer Registry, Pieni Roobertinkatu 9, 00130 Helsinki, Finland; 4 Public Health Evaluation and Projection, National Institute for Health and Welfare THL, Mannerheimintie 166, P.O. Box 30, 00271 Helsinki, Finland; 5 Department of Oncology, Tampere University Hospital, PL 2000, 33521 Tampere, Finland; 6 Present Address: Joint Research Centre (JRC), European Commission, Building 58A/006, Via Enrico Fermi 2749, TP 581, 21027 Ispra, Italy

**Keywords:** Disease-free time, Emotional non-expression, Patient-reported quality of life, Prostate cancer, Socioeconomic status, Stress, Psychological, Survival

## Abstract

**Purpose:**

The constructs evaluated in investigating association between psychosocial factors and cancer survival has varied between studies, and factors related to quality of life (QOL) have shown contradictory results. We investigated the effect of socioeconomic and early QOL and psychological factors on disease-free time and survival in localized prostate cancer.

**Methods:**

A consecutive sample of patients with localized prostate cancer (T1–3, N0, M0) treated with external beam radiotherapy completed validated questionnaires on coping with cancer (the Ways of Coping Questionnaire WOC-CA), anger expression (the Anger Expression Scale), life events (the Life Experience Survey), and various aspects of QOL (the Rotterdam Symptom Checklist, the Depression Scale DEPS, the EORTC QLQ-C30, the LENT-SOMA outcome measure) approximately 4.5 months after diagnosis. Cox regression analyses were used to determine the predictors of the disease-free and overall survival times measured from the date of diagnosis to the date of a PSA-relapse and date of death.

**Results:**

After controlling for biological prognostic factors, age, and adjuvant hormonal therapies, moderate and high socioeconomic status and an increased level of pain predicted longer survival, whereas an increased level of prostate-area symptoms and fatigue and, especially, reports of no/few physical symptoms were predictors of a shorter survival time. A longer PSA-relapse-free time was predicted by Cognitive Avoidance/Denial coping, whereas problems in social functioning, hopelessness, and an excellent self-reported QOL predicted a shorter PSA-relapse-free time.

**Conclusions:**

Higher socioeconomic status was prognostic for longer survival, as previously reported. Patients with a seemingly good QOL (few physical complaints, excellent self-reported QOL) had poorer prognoses. This association may due to the survival decreasing effect of emotional non-expression; patients with high emotional non-expression may over-report their wellbeing in simple measures, and thus actually be in need of extra attention and care.

## Introduction

The association between psychosocial factors and cancer survival has been convincingly demonstrated only during recent decades [1–12]. The psychological processes and constructs investigated have varied among studies [4, 8]. Many of the earlier studies were retrospective, biological prognostic factors were not sufficiently controlled for, and only certain factor(s) were investigated. In many of the prospective studies, the survival follow-up time was very short [4, 8–10, 13, 14]. In addition, socioeconomic factors were not often taken into account [4, 15, 16].

Studies suggest that some psychosocial factors, such as emotional expression and psychological stress, are more likely to have an effect on survival compared with other factors [4]. Specifically, emotional non-expression (“repression”) [6, 17–19], hopelessness [7, 20, 21], depression [1, 10, 11, 20], and stress-related psychological factors [8, 12, 20] have been suggested to predict a shorter survival, whereas social support [22] and denial or minimizing coping response to the cancer to predict a more favorable prognosis [2, 5–8, 12, 21]. Factors related to well-being and quality of life (QOL) have shown contradictory results [2, 5–7, 9, 23]. In some studies, a good reported QOL has shown an association with a poorer prognosis [6, 7, 9].

Among the number of studies investigating the impact of psychosocial factors on cancer progression, very few have dealt with prostate cancer. However, prostate cancer is the most common cancer among men in developed countries, including Finnish men (http://www.cancerregistry.fi). In most countries, the average age at prostate cancer diagnosis is greater than 70 years. The incidence is increasing worldwide in part due to the aging populations and the widespread testing for prostate-specific antigen (PSA) [24–27]. Today, most prostate cancers are detected when they are localized or at most locally invasive, and the prognosis is generally good [24–28].

A major limitation of many previous studies was that the effect of psychosocial factors has been investigated in isolation from other related factors or processes [4, 13, 29–31]; however, the effect of these factors does not occur in isolation. Investigating their impacts separately results in over-simplification and misleading results. In particular, connections between coping and personality processes are important [18, 32, 33]. Therefore, the effect of psychological and psychosocial factors and their relative impact can be identified only when several factors are investigated and analyzed jointly, adjusting their effects on each other.

The psychobiological mechanisms that are thought to have an impact on cancer survival are related to psychological stress [4, 8, 34, 35]. According to psychological stress theories, the effects of stress on health outcomes depend on how a person can cope with the stress, coping being the main mediator in the process from stressful events to outcomes such as psychological symptoms and somatic illness [36, 37]. Social support and personality factors modify the coping process [38]. In the process-oriented view [36, 37, 39], coping is defined as “the person’s constantly changing cognitive and behavioral efforts to manage specific external and/or internal demands that are appraised as taxing or exceeding the person’s resources.” There are no in built assumptions on good or bad coping. The actual coping process refers to the person’s efforts to reduce, minimize, master or tolerate the person–environment transaction that has been appraised to be demanding. Psychological stress processes have been linked with biological immune down-regulation in cancer [34].

According to a model proposed to guide research in psycho-oncology [33], the impact of cancer disease and treatment effects are mediated by personal (including personality and coping style, adjustment, and sociodemographic factors), medical, social-environmental, and life stressor factors [3, 12, 29, 33]. The outcomes of these pathways are QOL and survival. In particular, a personality-related coping response called non-expression of negative emotion is potentially associated with poorer cancer progression [4, 7, 17–19, 40]. Non-expression of negative emotion refers to a tendency to suppress (i.e., not to express) negative feelings [17, 32], such as anger and fear, and leads to less effective coping in stressful situations. It has been described as being emotionally contained and “maintaining a facade of contentment” in social relationships. Related concepts, such as emotional defensiveness (antiemotionality, i.e., suppression and control of emotions) has also been found to predict reduced survival in cancer [6, 17]. Furthermore, there is a suggested cancer-prone Type C response style [18, 19], which is a multidimensional construct that includes non-expression of negative emotions as a core element, where helplessness/hopelessness in stressful situations, and self-sacrificing, over-cooperative, and appeasing behavior are added. Type C response style has been found to be associated with reduced survival in cancer.

We previously presented a conceptual model on the psychosocial predictors of wellbeing of cancer patients [35]. In the model, we assume that both QOL and survival are outcomes in a stress process, which is mediated by psychological stress processes and initiated by cancer and treatment. Coping with cancer is the main mediator in the process, and social support and personality factors modify the coping process. Furthermore, we assume that these processes are interfered by non-cancer life stresses. We previously applied the model for studying baseline psychosocial predictors of survival outcomes in localized melanoma [7, 12] and breast cancer [6] patients under 72 years of age. We identified some gender differences, indicating that men may respond to different types of psychosocial factors compared with women [12].

In the present study, on the basis of the model and experiences from our previous studies, we investigated the baseline and early predictors of disease-free and overall survival times in prostate cancer patients of all ages and treated with external beam radiotherapy in Finland. We hypothesized that specific factors in the psychological stress processes (patterns of coping, anger expression traits, non-cancer life events), and components of patient-reported QOL evaluated at the time of primary treatment are associated with disease-free time and survival in localized prostate cancer.

## Methods

### Patients and procedure

Newly diagnosed T1–3, N0, M0 prostate cancer patients who were admitted for treatment to the Department of Oncology, Tampere University Hospital (Finland) in 2002 and were treated with curative external beam radiotherapy were consecutively included as eligible patients for the study (*n* = 104). Patients who were mentally and physically able to participate in a study interview and complete questionnaires (no active severe mental or neurological disease) and with no previous cancer disease^1^ were invited to participate; 6 patients were not invited because they could not be interviewed (3 dementia, 2 consequences of stroke, and 1 acute state of another physical illness), and 3 patients based on a previous cancer. Six patients refused to participate. In the end, 89 patients (86%) were interviewed. An additional 3 patients were excluded from the survival analyses because they had a T4 disease, and 5 patients because they had underwent radical prostatectomy prior to the radiotherapy. Thus, the final study group comprised 81 patients (Table 1).

**Table 1:** Demographic, socioeconomic, disease, and treatment variables in patients

Variable	*N* = 81 (%)	
Age, mean 66.5 years (median 68, range 51–82 years)		
50–59		10 (12)
60–69		48 (59)
70–79		22 (27)
> 80		1 (1)
Marital status		
Single		1 (1)
Married or cohabiting		68 (84)
Divorced		7 (9)
Widowed		5 (6)
Children, mean 2.3		
Have children		75 (93)
Vocational education		
None		33 (41)
Vocational courses		7 (9)
Vocational school		17 (21)
College		17 (21)
University education (any)		7 (9)
Yearly family income (EUR)		
< 17,000		19 (23)
17,000–25,000		22 (27)
25,000–34,000		19 (23)
34,000–42,000		10 (12)
> 42,000		11 (13)
Socioeconomic status (SES)		
Low		12 (15)
Moderate		57 (70)
High		12 (15)
Gleason classification (x + x)		
3–4	9 (11)	
5	9 (11)	
6	28 (35)	
7	21 (26)	
8	7 (9)	
9–10	4 (5)	
Unidentified	3 (4)	
Tumor classification		
T1	28 (35)	
T2	29 (36)	
T3	24 (30)	
Biological risk^a^		
Low	28 (35)	
Moderate	31 (38)	
High	22 (27)	
Radiation		
Total dose	Mean 69.9, range 68.0–70.4	
Radiation for pelvic lymph nodes	8 (10)	
Neo-adjuvant hormonal therapy		
None	28 (35)	
LHRH analog	53 (65)	
Adjuvant hormonal therapy		
Yes	34 (42)	
Other chronic disease/condition		
Yes	66 (81)	

The hospital district was ethnically homogeneous regarding the age groups in question

^a^According to nomogram for prostate cancer recurrence [50]: classified as ‘Low risk’ if Gleason ≤ 6 and PSA < 10, ‘Moderate risk’ if Gleason = 7 or PSA 10–20, and ‘High risk’ if Gleason ≥ 7 or PSA > 20

In Finland, hospital districts organize specialized medical care. Some specialized medical care services, e.g., oncology, are organized on the basis of special responsibility areas of the five university hospitals of Finland. Because of this centralized cancer care, nearly all patients diagnosed in the region and treated with external beam radiotherapy were included in the eligible patients.

The patients were interviewed at approximately 4.5 months (range 2–9) after diagnosis by the same interviewer (a medical student, the third author) during a visit to the Department of Oncology for external beam radiotherapy. A patient was not interviewed until two months after the diagnosis to avoid disturbing the patients in an acute cancer crisis and to achieve a more reliable measurement, and each patient was currently receiving external radiotherapy (65% with LHRH analog as neo-adjuvant hormonal therapy). Each interview took approximately 1.5–2.5 h. During the interview the patients completed several validated questionnaires addressing various psychological factors and aspects of patient-reported well-being and QOL (see, Measures, below). The patients were also asked to report their demographics, vocational education, and family income. Most of the measures were the same as used in our previous melanoma [7, 12] and breast cancer [6] survival studies, but two health-related QOL measures (the EORTC QLQ-C30 [41] and the LENT-SOMA outcome measure [42]) were added in the present sample.

### Measures

Cancer and treatment data (staging, Gleason classification, neo-adjuvant hormonal therapy, and adjuvant hormonal therapy; Table 1) and survival time and disease-free time data (date of diagnosis, date of death, date of PSA-verification of recurrence of the cancer) were obtained from hospital medical records.

Socioeconomic status was operationalized as a combination of vocational education and yearly family income (total income of the household). It was classified as ‘low᾽ if the patient had no occupational education and the yearly income was < 17,000 EUR, ‘moderate᾽ if the income was 17,000–34,000 EUR, and ‘high᾽ if the patient had a college or university education and/or the yearly income was ≥ 34,000 EUR (Table 1).

The measured psychological/psychosocial and QOL domains are listed below:

1. Coping with cancer was measured using *the Ways of Coping Questionnaire (WOC)*, a 50-item self-report questionnaire (scale 0–3 in every item) developed “to identify the thoughts and actions an individual has used to cope with a specific stressful encounter”—here any aspect of the prostate cancer since the diagnosis. We used an item structure proposed to form a cancer-specific WOC-CA measure [43, 44], including the patterns Focusing on the Positive, Distancing, Seeking and Using Social Support, Cognitive Avoidance, and Escape Avoidance.
2. The traits of anger expression were evaluated using *the Anger Expression Scale (AX Scale)* [45]. It refers to “the extent that an individual engages in aggressive behaviors when motivated by angry feelings” and taps three dimensions: Anger-in (“individual differences in the frequency that angry feelings are experienced but held in”), Anger-out (“...feelings of anger are expressed in aggressive behavior”), and Anger control (“...an individual attempts to control the outward expression of angry feelings”).
3. Stressful life events were recorded from the preceding year using *the Life Experience Survey (LES)* [46], a list of 50 events addressing both the number of life events and their perceived impact.
4. To measure patients’ symptoms and their intensity, we used *the Rotterdam Symptom Checklist (RSCL)* [47], which has been developed to measure symptoms reported by cancer patients. The RSCL includes 30 symptoms (8 psychological and 22 physical, scale 0–3 in every symptom) that the patients may have experienced during the past week and a separate single item index on overall global quality-of-life with scale from 1 (extremely poor) to 7 (excellent). The level of depressive symptoms was measured using *the Depression Scale DEPS* [48], which has been developed for screening of depression in primary health care settings and evaluates 10 feelings and depressive symptoms (scale 0–3) with a coverage-period of the previous month. One of the DEPS symptoms is hopelessness.
5. Health-related QOL of the patients was measured using *the European Organization for Research and Treatment of Cancer Quality of Life Questionnaire (EORTC QLQ-C30)*^2^ version 3.0, Finnish translation [41]. The measure is divided into a global health status scale, five functional scales (Physical/Role/Emotional/Cognitive/Social functioning) and several symptom scales/items, e.g., fatigue, nausea, and pain; all evaluated from the previous week. Local prostate-area symptoms were evaluated using a modification of the *LENT-SOMA outcome measure* (see Footnote 2) [42], a clinical tool developed to record and score normal tissue effects of radiotherapy (urinary, bowel, and sexual symptoms and dysfunction).

The study protocol was approved by the ethical committee of Tampere University Hospital, and informed consent was obtained from each participant. The first author is bound by national (The Union of Finnish Psychologists) and international (American Psychological Association) ethical codes of psychology.

### Statistical analyses

Descriptive statistics, ANOVA, *t* test, and Pearson’s correlation (*r*), were used to explore the sample. Regression analysis was used to investigate the association between background factors, psychological/psychosocial factors, and the QOL indicators. The Cox proportional hazards regression model [49] was used to determine the simultaneous and relative contribution of the socioeconomic, psychological, and QOL predictors on disease-free and overall survival times, controlled for age, biological prognostic factors, and hormonal treatment. The predictor variables were separately tested for both outcomes. Predictors of the survival times in the Cox models were considered significant if the corresponding *p*-value was < 0.05.

Overall survival was measured from the date of diagnosis (date of PAD) to the date of death and disease-free time from the date of diagnosis to the date of a PSA-verified relapse, or the survival was censored at the date of last follow-up (March 31, 2011). A biological risk classification into mild, moderate, and severe for T1–2 tumors [50] (Table 1, footnote a) and separately T3 tumors was used as a biological prognostic factor. Implementation of neo-adjuvant hormonal therapy and adjuvant hormonal therapy were used as prognostic treatment factors.

In the Cox models, age, biological prognostic factors, and treatment (the hormonal therapies) were always included in the models. Second, we added the socioeconomic status variable(s) to the models to investigate their impact, and adjust for their effect when investigating the effects of the other variables. Third, the psychological and QOL indicators (see Measures; selected on the basis of the theoretical model [35]) were added into the models individually and in various combinations, and following experiences from our previous corresponding studies. To avoid testing of related variables, we investigated their psychometric properties and mutual associations, and selected the variables that were the best (high Cronbach’s Alpha, symmetrical distribution) and were least correlated with each other. In some variables, we divided the answer options into two categories (no/yes). The proportional hazard assumption was tested [51] for specific variables and globally. Statistical analyses were performed using SPSS for Windows 18 and 20, and Stata 11 (StataCorp. 2013. Stata Statistical Software: Release 13. College Station, TX: StataCorp LP, USA).

## Results

By April 2011, eighteen (22%) of the 81 patients had died, and the follow-up time was ≥ 8 years 4 months (median 8.54 years, 95% CI 31.01–31.25 days; no patients were lost from the overall survival follow-up). Information on progression of the prostate cancer, i.e., PSA follow-up was available for 77 patients, and 18 (23%) of them had been diagnosed with PSA verification of prostate cancer relapse. Five patients with relapse had died.

### Association between the psychological and QOL variables

According to our theoretical model [35] (see, Introduction), we investigated first the association between the potential predictor variables (background and disease/treatment variables, anger expression, and coping) and the wellbeing/QOL indicators. All the QOL indicators were strongly intercorrelated (*p*-values generally < 0.001). Therefore, we separately investigated predictors of the RSCL and the EORTC QLQ-C30 subscales, and the DEPS. On the whole, the disease and treatment variables did not associate with QOL, whereas the coping and anger expression variables were associated with several wellbeing and QOL indicators. Higher income of the family predicted more psychological symptoms and problems in Role functioning, Emotional functioning, and Cognitive functioning. Escape coping was associated with a poorer and Distancing coping with a better psychological health and self-perceived QOL, as measured by all the indicators. The Anger-in trait was associated with poorer wellbeing, whereas Anger control was associated with better wellbeing when evaluated by any of the measures despite social functioning.

### Predictors of overall survival and disease-free time

T3 tumor (HR 5.51, 95% CI 0.88–34.45, *p* = 0.07) and neo-adjuvant hormonal treatment (HR 0.26, 95% CI 0.05–1.31, *p* = 0.1) were only weakly associated with overall survival. Disease-free time was not significantly predicted by any of the disease and treatment variables.

### Individual associations between the predictor variables and survival outcomes

Lack of vocational education showed an individual association with both shorter survival and disease-free times, and low family income was associated with shorter survival (Table 2). Some indicators of a good QOL were individually associated with poorer outcomes: low level of reported physical symptoms (the lowest 18.5% vs. the remaining) was associated with both shorter survival and disease-free times, and the highest score in the single-item quality-of-life index (‛excellent’, reported by 18.2%) was associated with a shorter disease-free time. Furthermore, increased pain and reported number of life events were individually associated with longer survival.

**Table 2:** Measures with statistically significant individual associations with overall survival time and disease-free time

Individual predictors	Univariate analyses			
	Association with overall survival time		Association with PSA-relapse-free time	
	HR (95% CI)	*p*	HR (95% CI)	*p*
No vocational education	2.78 (1.02–7.59)	0.046	2.40 (0.89–6.46)	0.083
Family income low (< 17,000)	4.85 (1.69–13.90)	0.003	n.s	
Level of physical symptoms low, no/yes^a^	5.99 (2.02–17.77)	0.001	3.21 (0.96–10.72)	0.058
Self-reported QOL ‘excellent’, no/yes^b^	n.s		5.21 (1.81–14.95)	0.002
Pain^c^	0.20 (0.05–0.83)	0.026	n.s	
No. of non-cancer life events^d^	0.71 (0.51–1.00)	0.048	n.s	

*HR* hazard ratios in Cox models: impact of each predictor analyzed individually and adjusted for age, biological prognostic factors and adjuvant hormonal therapies

^a^Physical symptoms scale (RSCL) low = score < 7 (18.5%) versus the remaining scores. When the scale was divided into two levels based on the median, the higher level was associated with a longer overall survival with HR 0.43, *p* = 0.1

^b^In the RSCL quality-of-life index (1–7) the highest score 7 ‘excellent’ (18.2%) versus the remaining scores

^c^A scale in the EORTC QLQ-C30

^d^By the LES

### Simultaneous impacts of the predictor variables on overall survival

Patients’ moderate and high socioeconomic status predicted longer overall survival when adjusted for the biological and treatment variables, with multivariate hazard ratios of 0.16 (95% CI 0.05–0.46, *p* = 0.001) and 0.11 (95% CI 0.02–0.6, *p* = 0.015), respectively.

When the psychological and QOL variables were added to the model, we found that a complex combination of variables predicted overall survival (Table 3). Different QOL measures exhibited either a favorable or an unfavorable impact, i.e., an increased level of pain (HR 0.05; 95% CI 0.01–0.32) predicted longer survival, whereas prostate-area symptoms (HR 1.18; 95% CI 1.03–1.36), increased fatigue (HR 7.08; 95% CI 1.77–28.32), and reports of no or few physical symptoms (HR 9.90; 95% CI 1.48–66.30) were significant predictors of shorter survival time. However, when the overall quality-of-life index (total scale 1–7) was tested instead of the prostate-area symptom scale, it predicted a longer survival (HR 0.51, 95% CI 0.27–0.95, *p* = 0.033); when both scales were included, the effect of the overall QOL was weaker (HR 0.56; 95% CI 0.27–1.15, *p* = 0.113).

**Table 3:** Psychosocial and QOL measures at the time of curative radiotherapy predicting overall survival

Predictor	Multivariate analysis	
	HR (95% CI)	*p*
Age, years	0.98 (0. 89–1.07)	0.599
Biological risk		
Moderate risk in Tumor 1–2	0.15 (0.02–0.97)	0.047
High risk in Tumor 1–2	0.52 (0.06–4.64)	0.562
T3 Tumor	4.98 (0.63–39.29)	0.127
Neo-adjuvant hormonal therapy, no/yes	0.17 (0.02–1.21)	0.076
Adjuvant hormonal therapy, no/yes	1.54 (0.24–9.77)	0.649
Socioeconomic status		
Moderate	0.20 (0.04–0.91)	0.037
High	0.04 (0.01–0.34)	0.003
Level of physical symptoms low, no/yes^a^	9.90 (1.48–66.30)	0.018
Pain^b^	0.05 (0.01–0.32)	0.002
Urinary, bowel, and sexual symptoms ^c^	1.18 (1.03–1.36)	0.017
Fatigue^b^	7.08 (1.77–28.32)	0.006

*HR* hazard ratios in the Cox model; all variables were adjusted for each other

^a^Based on the RSCL Physical symptoms scale: the lowest 18.5% versus the remaining scores

^b^A scale in the EORTC QLQ-C30

^c^The LENT-SOMA scale. When the RSCL overall quality-of-life index (1–7) was tested instead of the LENT-SOMA prostate-area symptoms, its relative HR was 0.51 (95% CI 0.27–0.95, *p* = 0.033)

### Simultaneous impacts of the predictor variables on PSA-relapse-free time

The PSA-relapse-free time was not significantly predicted by any of the biological prognostic factors, hormonal treatment, or socioeconomic status. A longer disease-free time was predicted by Cognitive Avoidance/Denial coping (HR 0.76; 95% CI 0.59–0.97), whereas a shorter relapse-free time was seen for patients with problems in social functioning (HR 3.32; 95% CI 1.45–7.56), hopelessness (HR 8.90; 95% CI 1.62–48.87), and an excellent self-reported QOL (HR 47.31; 95% CI 6.35–352.33) (Table 4).

**Table 4:** Psychosocial and QOL measures at the time of curative radiotherapy predicting PSA-relapse-free time

	Multivariate analysis	
	HR (95% CI)	*p*
Cognitive avoidance/denial coping^a^	0.76 (0.59–0.97)	0.031
Self-reported QOL ‘excellent’, no/yes^b^	47.31 (6.35–352.33)	< 0.001
Hopelessness, no/yes	8.90 (1.62–48.87)	0.012
Problems in social functioning^c^	3.32 (1.45–7.56)	0.004

*HR* hazard ratios in the Cox model; all variables were adjusted for age, biological risk, hormonal therapies, and SES (which were all statistically non-significant), and were adjusted for each other

^a^Cognitive avoidance coping was statistically significant only when all the other variables were included in the model

^b^Based on the RSCL quality-of-life index (1–7): the score ‘excellent’ (= 7) versus the remaining scores

^c^The EORTC QLQ-C30. When Anger-in personality trait was tested instead of the social functioning, it showed HR of 1.15 (95% CI 0.98–1.37, *p* = 0.1)

The proportional hazard assumption was successfully tested for the final models locally and globally using Schoenfeld residuals [51], indicating that these impacts remain stable over the follow-up period.

## Discussion

Overall survival and disease-free time in patients with localized prostate cancer were predicted by socioeconomic status, psychological factors, and patient-reported QOL. Different QOL measures demonstrated contradictory results as either a favorable or an unfavorable impact. Surprisingly, we found that the patients’ reports of no or few physical complaints predicted shorter survival, whereas reported pain was prognostic for longer survival. In addition, a longer PSA-relapse-free time was predicted by responding to the cancer diagnosis with a Cognitive Avoidance/Denial coping pattern, whereas problems in social functioning, hopelessness, and—contradictorily—reporting an excellent QOL, predicted a shorter PSA-relapse-free time. We suggest an explanation for these surprising findings, i.e., that the observed survival decreasing effect of high scores in certain simple QOL measures may reflect a trait of non-expression of negative emotions (a tendency not to express negative feelings and to keep ‘facade of contentment’), that has been found potentially associate with cancer progression. We propose that the patients who scored exceptionally low/high in simple wellbeing/QOL measures may have been those with high non-expression of negative emotions and that they thus over-reported their wellbeing.

Few earlier studies on psychosocial factors and cancer survival have addressed prostate cancer [8, 52, 53] or male patient samples that also include the oldest age-groups. We were able to collect a relatively small but regionally representative sample of newly diagnosed prostate cancer patients. At the time of the data collection, the prostate cancer therapy was different than today (e.g., active surveillance was hardly used) and the active treatment modes have improved ever since. There were no population PSA-screening programs going on in Finland. The study sample was limited to patients who underwent curative radiotherapy, and the other major definite therapy mode, radical prostatectomy, was not represented. However, younger and healthier patients more often undergo operation, whereas external radiation is received by a wider patient cohort [54]. For practical reasons, the timing of the measurement since diagnosis varied (2–9 months); the onset of the radiotherapy differed, and the patients were willing to take part to the interview at different time points during their radiotherapy.

We investigated both overall survival and disease-free times because they are very different outcomes in cancer types with good prognoses, such as prostate cancer, and may thus be predicted by different mechanisms; we found that they were indeed predicted by different factors. The potential predictors were simultaneously investigated to account for their relative impact, and were either derived from a theoretical model [35] or were previously demonstrated to exhibit an association with cancer survival [4, 20, 33]. Most of the predictor variables were subscales of established validated questionnaires. The radiotherapy was currently going on in all patients. The analyses were carefully adjusted for age, known biological prognostic factors, and treatment.^3^

We found that a higher socioeconomic status predicted a longer survival both independently and when evaluated with the psychological and QOL factors. The survival prolonging effect of socioeconomic status in cancer is well known [15, 16, 55], and has been previously reported in prostate cancer patients receiving curative treatment [53]. However, previous studies often considered its effect alone without other potential predictors [52, 53, 56]. We concur with previous studies suggesting that socioeconomic status should be taken into account to obtain valid information on the impact of QOL on survival in prostate cancer [57].

The measures associated with poorer outcomes appeared to be the simple indicators of self-reported wellbeing, i.e., low scores in perceived physical symptoms and an excellent score in an overall QOL index. We did not find negative association between established QOL measures and the survival outcomes, or if there was an association, it was positive. We suggest that these results may reflect the harmful effect of non-expression of negative emotions, which has been defined to refer a tendency not to express negative feelings and to keep ‘facade of contentment’[17]. According to this theory, individuals with high non-expression of negative emotions potentially under-report their symptoms as they are prone to a repressive coping style, i.e., tend to avoid a negative affect, and consequently answer wellbeing measures overly positively [17, 32]. This idea was supported by the finding that the Anger control personality trait was associated with less reported symptoms and a better QOL.

One supporting factor to our argument is that scoring low in symptom measures and very high in overall QOL at the time of recent cancer diagnosis and ongoing radiation treatment may not reflect the true objective situation. Instead, these scores may reflect a personality trait or coping behavior at a stressful situation (recent cancer diagnosis, ongoing radiotherapy). Furthermore, when e.g., the quality-of-life index was tested as a total scale (very poor–excellent) it predicted a longer survival. So, our results did not indicate that reporting a good QOL was hazardous, but that reporting an ‘excellent’ QOL was. Individuals with a repressive coping style have also previously been found to score low in self-reported physical symptom scales [58]. Up to 20% of the population has been found to answer self-report scales in an overly positive fashion [32], which corresponds with our finding that the hazardous effect on survival was identified when the scores were divided into two categories: no/very few symptoms or an ‛excellent’ QOL versus other (both were present in approximately 18% of the patients).

The results obtained on simple wellbeing/QOL indicators support the conclusions we have made in our previous studies [6, 7], i.e., that simple global-rating methods are not suitable of eliciting information on QOL of all cancer patients. Certain subgroups (“repressors”) seem to understate their condition and over-report their wellbeing. There is a risk that the simple global-rating measures actually provide more information on non-expression of negative emotions behavior than QOL. It has been suggested [32] that to elicit information from repressors measures requiring specific answers should be used instead of single-item indexes or symptom lists.

Prostate-area symptoms (urinary, sexual, bowel) and fatigue—as evaluated with well-established validated measures comprising specific questions—were prognostic for shorter survival, as expected. These are common adverse effects in external radiation and are likely to be well recorded at the oncology clinic, and patients may be less likely to under-report them. This measurement was, however, not congruent because the timing of the interview during the radiotherapy varied, which potentially affects the level of side effects. However, the survival model also held when the overall QOL index (total score 1–7) was tested instead of the prostate-area symptoms. These factors both indicated the wellbeing/ill-being of the patient during the primary treatment. Self-evaluation of the wellbeing status is continuously presented to be a reliable indicator of an individual’s current and future health.

The obtained results on problems in social functioning and hopelessness, and a shorter relapse-free time may also relate to concepts close to non-expression of negative emotions, such as antiemotionality (“emotional defensiveness”) [17] and Type C response style [18, 19]. Antiemotionality (suppression and control of emotions) is manifested in self-sacrificing behavior, which may eventually lead to problems in social functioning. Helplessness/hopelessness in stressful situations is a component of the Type C style. We previously found that antiemotionality predicted shorter survival in breast cancer [6] and hopelessness in melanoma [7]. Hope at cancer diagnosis is essential [59] and it may predict survival [1, 11]. Hopelessness has been found to affect cancer mortality at the population level [60], especially in older populations [61], to which most prostate cancer patients belong. Moreover, the observed favorable effects of pain and number of stressful life events may be related to non-existence of non-expression of negative emotions: those without may have reported more symptoms and events.

In accordance with our previous studies [6, 7, 12], we identified only one protective psychological factor: the Cognitive Avoidance/Denial coping pattern, and it predicted only the disease-free time. As noted above, overall survival and PSA-relapse-free time are very different outcomes in prostate cancer; the former is less affected by the cancer because of the good prognosis, whereas the latter is cancer-specific. This result is in line with cumulating research suggesting that a favorable prognosis in cancer is predicted by responding to the cancer diagnosis by a coping pattern of denying [21] or, nowadays more often called, ‘minimizing’ the fact of having cancer [1, 2, 4–7, 17, 20, 29]. This concept refers, according to definition of coping, the patients’ efforts to manage demands caused by the cancer by minimizing impact of the disease, not the consequent negative affect. However, denying/minimizing may at a later point of time have a different effect [59].

Our results provide an explanation for why good self-reported QOL in some previous studies appeared to have had an unfavorable impact on cancer survival outcomes. Patient reports of a good wellbeing in simple measures may reflect non-expression of negative emotions. Thus, very high reports of wellbeing in newly diagnosed cancer patients may indicate a psychological risk factor for reduced survival outcomes. It may be good for the patients to have complaints at the time of primary treatment. This finding may have clinical relevance given that patients who report no or few problems (no symptoms, excellent QOL) may actually be vulnerable and in need of extra attention and care. However, more research is needed, e.g., to investigate the issue in patients with different cancer types/different diseases, in both genders, in different phases of care and rehabilitation, and in prospective designs.

Clinicians are to interpret scores of validated questionnaires on wellbeing and QOL as usual. A clinician who interprets the scores should know the patient’s situation, e.g., phase of treatment and time since diagnosis. Conclusions of the patients’ wellbeing should not be based on a single measurement. Validated questionnaires are good tools for screening of state of QOL, which may require further clinical examination. Our criticism is targeted to simple QOL indicators which are easily influenced by other factors, e.g., non-expression of negative emotions.

## Footnotes

1 A previous cancer was anticipated to influence the psychological stress processes that were the initial research target.

2 The EORTC QLQ-C30 and the LENT-SOMA outcome measure were not used in our previous survival studies.

3 As expected, having a locally invasive tumor (T3) predicted a shorter survival when compared with fully localized disease, and neo-adjuvant hormonal therapy was effective.
